# Neuropeptide immunofluorescence in human nasal mucosa: assessment of the technique for vasoactive intestinal peptide (VIP)

**DOI:** 10.1016/S1808-8694(15)31299-4

**Published:** 2015-10-20

**Authors:** Jeferson Cedaro de Mendonça, José Eduardo Lutaif Dolci

**Affiliations:** ^1^ Master degree (physician); ^2^ Joint Professor, Department of Otorhinolaryngology, Santa Casa de Sao Paulo

**Keywords:** vasoactive intestinal peptide, neuropeptides, nasal mucosa, immunofluorescence

## Abstract

Neuropeptides are important neurotransmitters in nasal physiology and the increasing knowledge of their role in nasal diseases brings new therapeutic perspectives. The investigation of human nasal mucosa neuropeptides is based mostly on immunocytochemistry, a complex approach whose resulting factors may be variable. Aiming to make this kind of research available, an immunofluorescence approach for vasoactive intestinal peptide (VIP) in human nasal mucosa is proposed and evaluated.

**Study design:**

Transversal cohort.

**Material and Method:**

Human inferior turbinate samples were obtained at time of nasal surgery from eight patients. The samples were fixed in Zamboni solution (4% phosphate-buffered paraformaldehyde and 0.4% picric acid), snap-frozen and stored at -70ºC. 14 µm sections were then obtained. Immunofluorescence staining for VIP (Peninsula Laboratories) was performed and its images documented by conventional photography. The method's specificity, sensitivity and reproducibility of execution were evaluated. Additionally, the reproducibility of interpretation of results was evaluated through the comparison of staining scores (0 to 4) attributed to the images by six observers.

**Results:**

The results showed the approach to be very specific and sensible, besides being reproducible in its execution. The interpretation of results may depend on the observer's accuracy in judging immunofluorescence images, but it showed uniformity.

**Conclusion:**

The proposed method was highly useful for research purposes in neuropeptides in human nasal mucosa.

## INTRODUCTION

The role of nose in protecting the airways, through its classical functions of filtration, heating and humidification of inhaled air, is effective thanks to the peculiar anatomy of the nasal cavity and functional aspects of the lining mucosa. The capability to widely vary its volume, modifying the air resistance by congesting venous plexus, in addition to large variations of gland secretion according to exogenous and endogenous factors, are intriguing phenomena of the nasal physiology that are continuously studied^1^. The role of abundant sympathetic, parasympathetic and sensitive innervation in the control of these phenomena is extremely important^2^.

The balance of classical neurotransmitters, acetylcholine and noradrenalin, present in sympathetic and parasympathetic innervation - and sometimes in their imbalance - have been predominant in the explanation of nasal physiological and pathological phenomena, and they have guided the therapeutic principles of nasal pathological conditions. Together with sympathetic and parasympathetic innervation, sensitive innervation has a significant physiological role in the protection reflex of airways, regulating the blood flow and consequently, the nasal air resistance, glandular secretion, and also mediating inflammatory and allergic responses^2^, ^3^.

The discovery and study of new neurotransmitters, the neuropeptides, in different animal tissues and also in human beings have brought to light new concepts about physiology of innervation of these organs. There are dozens of neuropeptides already being studied, and the respiratory system is widely investigated^4^.

Neuropeptides coexist with classical neurotransmitters, playing a key role in modulation. Generically, the neuropeptide action is more subtle, but it is long-lasting, in addition to the interaction with cells and allergic and inflammatory mediators, which have a more complex function when compared to classical neurotransmitters, which impact the study of nasal physiology and pathophysiology. Considering the neuropeptides, substance P (SP), neurokinin A and B (NKA and NKB), calcitonin gene related peptide (CGRP), gastrin release peptide (GRP), vasoactive intestinal peptide (VIP), peptide histidin methyonine (PHM) and neuropeptide Y (NPY) have been studied in the respiratory system^5^.

Therefore, the demonstration of these neuropeptides in human nasal mucosa concerning their presence, epithelial distribution and lamina propria structures, in addition to differences between normal and pathological conditions, has been the object of research and brought light to deep understanding of nasal physiology and pathophysiology^2^, ^6^. The progression of this knowledge may point towards new alternatives for nasal disease treatment in the near future, thanks to the development of antagonists, considering that there are already some results in central nervous system^7^.

However, so that it may become a fact, we need to have concepts and conclusions based on studies with large samples and preferably, coming from different universally distributed research centers. Only then we will be able to define facts about the actions of neuropeptides in human nasal mucosa that can really reflect clinical practice applications. The methodology used in the study of neurotransmitters concerning presence and distribution of tissues is normally based on immunelabeling, which is a complex technique that has variations and is relatively expensive. The identification of neuropeptides brings special technical difficulties owing to the fragility of molecular structure and easy degranulation and elimination of nervous fibers, suffering the action of proteolytic enzymes, requiring a detailed technique, from collection of tissue to be studied to its processing^8^. All neuropeptides listed here are located inside nervous fibers that are distributed in the human nasal mucosa, so as that when they are identified by immunelabeling, they can delineate the fiber they contain^9^. The fact that neuropeptides belong to a specific molecular class and share physiological similarities allows the use of a common technique of identification concerning aspects of tissue manipulation, reaction of immunelabeling and observation of results, ranging exclusively from neuropeptide specific antibodies in question (primary antibody), which is provided by reaction kits. In this study, we conducted immunofluorescence reaction for VIP, which is one of the most important neuropeptides, present in nervous fibers that are distributed on the lamina propria of human nasal mucosa^6^, ^10^. There are no studies on immunelabeling of neuropeptides on the human nasal mucosa published in our country. Thus, our paper intended to find the domain and standardization of an immunofluorescence technique for human nasal mucosa neuropeptides, providing an important investigation method to be reproduced and uniformly interpreted to support further investigations in nasal physiology and pathophysiology.

## LITERATURE REVIEW

### Neuropeptides

Neuropeptides are neurotransmitters present in neurons and nervous fibers spread throughout the whole body, also found in experimental animal models. They are peptides, or sequence of amino acids synthesized in cell bodies and transported to axon endings. When classical neurotransmitters are released, they demonstrate some differences: they are released in regions not-exclusively synaptic, suffer enzymatic degradation after release, not used for reuptake, renovation takes place exclusively by synthesis of cell body, and it is a slower process compared to classical neurotransmitters, which can be recaptured in synaptic chink and immediately reused^4^.

In the nasal mucosa, SP, together with NKA, NKB and CGRP, form the group of sensorial neuropeptides abundantly found in cell bodies of trigeminal ganglion and nasal mucosa of animals and humans, distributed from the epithelium to glands and vessels^11^. They are vasodilators, and SP is the most powerful and CGRP has the longest effect^12^.

SP promotes increase in vascular permeability, leading to increase in concentration of proteins in nasal secretion^13^. This effect is exacerbated in patients with seasonal allergic rhinitis and it is not reduced with previous treatment with anti-histaminic, leading authors to conclude that SP effects would be independent from histamine release^14^. In a culture of human nasal mucosa cells, however, other authors confirmed the release of histamine based on the stimulation of SP^15^.

SP can have chemotaxic effect over neutrophils and eosinophils in subjects with allergic rhinitis, because there is increase in migration of cells and phagocytosis of nasal mucosa after administration of SP in periods of pollenization^5^.

Other significant action of SP and NKA in respiratory system is non-cholinergic bronchoconstriction. In humans, inhalation of SP and NKA causes bronchoconstriction only in asthmatic subjects, eliminated by sodium chromoglycate^16^.

GRP is a peptide found in nervous fibers and distributed on nasal mucosa similarly to SP, NKA and CGRP, probably in sensitive fibers, referred together with these neuropeptides, but its location is still unknown. The action seems to be related with secretion and not vascular phenomena^10^.

The main neuropeptide of parasympathetic innervation is VIP, found in sphenopalatine ganglion of rats and parasympathetic fibers that are distributed in nasal mucosa of animals and humans, coexisting with acetylcholine^5^, ^17^. VIP has vasodilating action observed in in vivo experiments in nasal mucosa of animals. In humans, there are in vitro experiments that demonstrated its vasodilating action in pulmonary tissue^17^.

Its presence in nervous fibers around the glands especially refers to the physiology of secretion. In the culture of human nasal mucosa cells, the increase of serous cell secretion after administration of VIP was observed, in addition to evidence of maximization of effect of cholinergic stimulation^18^, but there are studies that demonstrated secretion inhibitory action^10^.

We observed increase in concentration of VIP (in addition to SP and CGRP) in nasal secretion of allergic subjects sensitized after nasal provocation. The same authors also confirmed increase in VIP after administration of histamine^19^. There was higher density of immunereactive fibers to VIP in patients with allergic rhinitis when compared to normal subjects or those with hypertrophic rhinitis^3^.

NPY is present in sympathetic nervous terminations that are distributed in the respiratory system, including humans' nasal mucosa, coexisting with noradrenalin, with great density in thicker-wall vessels. NPY is a powerful vasoconstrictor, its action has more latency at first, but it is more long-lasting than noradrenalin. These findings suggest a significant role in control of blood nasal flow and nasal cycle^10^.

### Immunelabeling of neuropeptides

Immune, immunecytokine or immunohistochemical labeling is a method of location of an antigen that forms cell or tissue, using labeled antibodies, based on high affinity and specificity that characterizes the antigen-antibody reaction^8^. Depending on the type of marker linked to the antibody, the method is specifically named, such as for example: fluorescent markers - immunofluorescence; enzymatic markers such as peroxidase - immuneperoxidase.

Fluorescent markers, such as fluorescein isothiocyanide (FITC) were the first ones to be used; to visualize it, dark field microscope with appropriate filters are required. FITC, when excited by 490 nm wavelength, generates light green fluorescence^20^. One of the uses of immunofluorescence is location of nervous fiber neuropeptides, and in thick sections, with appropriate fixation, it allows the observation of sinuous fiber through the studied tissue^9^.

A disadvantage of immunofluorescence is that labeling is not permanent, because most of the markers tend to lose fluorescence with time, especially under the action of light^8^.

Tissues fixed in formalin tend to be auto-fluorescent and if they contain catecholamines, they may be induced and generate specific fluorescence colored close to fluorescein^21^.

Neuropeptides may suffer degranulation and elimination of fibers by chemical and mechanical stimuli. The technique for collection of fragment could influence the presence of neuropeptides by the use of topical medication (anesthetics and vasoconstrictors) and by mechanical manipulation, influencing the results of immunofluorescence^5^. The collection of nasal mucosa from inferior and medium concha is referred in the literature without technical details^2^, ^3^, ^6^, ^22^.

The method of tissue fixation is crucial for the success of immunelabeling. There is not an ideal method because both antigen and tissue require a specific type of fixation. Good preservation of the architecture of the tissue allows that the antigen be located in its biological context, and it is one of the main characteristics that mark the relevance of the immunelabeling method^8^.

There are two types of fixation solutions more frequently used in the preparation of histology slides: aldehydes, which promote food tissue preservation but may affect the antigen-capacity of peptides by immunofluorescence, and precipitates, that affect less the antigen structure of peptides, but may not manage to prevent dispersion^23^.

Immunofluorescence, with mixed fixation solutions, such as Zamboni solution (picric acid and buffered paraformaldehyde) are indicated because they balance these aspects^24^.

pH, fixation time and temperature may also influence the grade of tissue fixation and may impair antigenicity of peptides^25^, and many literature studies reported variation in these technical aspects^2^, ^3^, ^6^, ^22^.

The tissue may be fresh frozen, or in other words, without fixation process to conduct immunofluorescence, but the structure is not well preserved when compared to material with fixation. Conversely, the problems presented by the fixing solutions are solved.

However, manipulation of tissue not previously fixated require care, because the constituent proteins have higher chances of being spread^8^. Regardless, immediate fixation or tissue freezing before proteins are solubilized during decomposition process is essential for the objectives of immunelabeling^23^.

Ideal storage for immunofluorescence is freezing, because the inclusion in paraffin forms a barrier against penetration of antibodies^26^. The freezing technique, storage temperature and thickness of cryostat sections range in studies that described immunofluorescence for neuropeptides in human nasal mucosa^2^, ^3^, ^6^, ^22^.

The frozen specimen, if excessively dried, may hinder cryostat section, and we should be careful when storing it to eliminate air from neighboring tissues^25^.

The preparation of slides require cure concerning compliance to section. There are commercially available kits to this purpose, and previously prepared slides. For immunofluorescence applications, these elements are important because of the long reaction and exposure to rinsing^8^.

The documentation of slides using microscopic photography presents some difficulties concerning instability of fluorescence^8^.

Immunofluorescence serves primarily for qualitative information. However, some authors developed methods for its quantification, automated or not, that may allow comparisons^3^, ^6^, ^22^.

Once checked the importance of neuropeptides in human nasal mucosa and complexity and variability of immunofluorescence techniques in study, the purpose of the present study was to study an immunofluorescence technique to VIP to analyze its application for research purposes.

## MATERIAL AND METHOD

We collected nasal mucosa fragments of 8 patients submitted to nasal surgeries for nasal obstructions: septoplasty, turbinectomy and rhinoseptoplasty. The present study was approved by the Research Ethics Permanent Committee for Studies in Human Beings, State University of Maringá.

Authorization for the collection of material in the informed consent term was signed by the patients or responsible person.

We excluded patients that had high surgical or anesthetic risks.

There was no minimum or maximum age for inclusion.

Patients were instructed to interrupt antiinflammatory, hormonal or non-hormonal medication use, in addition to anti-histamine, systemic or topical, or any other drug or substance that could have influenced the results at least one week before the procedure, provided that there were no contraindication for their suspension. After collection, we performed the surgical procedure, whose technique and dressing did not differ from routine procedures.

Postoperative follow-up, care and drugs did not interfere in the habitual routine for this type of surgery.

Fragments were collected without nasal mucosa infiltration, but with topical vasoconstriction with adrenalin 1:10,000 for 5 minutes, with patients submitted to general anesthesia, without use of inhalation induction. Similarly, we tried to avoid excessive manipulation in the collection, which was facilitated by topical vasoconstriction and minimization of bleeding. We collected a fragment of inferior nasal concha from each patient, measuring about 1.0cm from its anterior extremity, 1 to 2.0cm long by 0.3 to 1.0cm width and variable thickness on the side that presented easy access.

Next, we describe the fixation procedures and storage:

Immediate fixation in Zamboni solution (paraformaldehyde 4% and picric acid 0.4% in phosphate buffer), for 6 hours at 4ºC; repetitive rising with saline buffered phosphate 0.1 M pH 7.4 (PBS), for 12 hours; cryoprotection in solutions of sucrose 18% in PBS 0.1M, for 24 hours; freezing in liquid nitrogen, after they had been soaked in frozen tissue liquid solution (O.C.T. 4583 compound - Tissue-Tek); stored in the freezer at -70ºC. Later, we made serial sections of 14µm with cryostat and placed them on previously prepared slides with organocylane adhesive 2% in ketone and stored at -1ºC. To observe histology structure of nasal mucosa and preservation of tissues through the fixation method used, from each of the four sections for immunofluorescence we collected two sections for hematoxylin-eosin staining (HE). For each fragment of nasal mucosa collected, we finally had eight sections for immunofluorescence and four for HE. Sections for immunofluorescence were placed on two slides, totaling 16 at the end.

### Immunofluorescence

To perform the technique, we acquired a kit for immunofluorescence for VIP: Immunofluorescence kit for VIP (human, porcine, rat) Peninsula Laboratories, code PENI - IFK7161.

Reactions were performed in two sessions, with four cases each, in different weeks. Next, we can see the steps of reactions:

#### Day 1:

Slides were brought to room temperature and rinsed for 3 times, during 10 minutes each, with phosphate buffer saline (PBS) 0.1M, then washed; we applied 200µl of goat serum (1:10) over the slides, and they were incubated at room temperature for 30 minutes in cold chamber. Slides were rinsed and we applied 200µl primary antibody (1:200) or negative control and slides were incubated at 4ºC for 24 hours in the same chamber.

#### Day 2:

We made three 10-minute rinses each with PBS 0.1M; slides were washed and excessive moist was dried with absorbent towel. We applied 200 µl secondary antibody (1:100), slides were incubated at room temperature for 60 minutes in the same chamber. We performed 3 washes for 10 minutes with PBS 0.1M; slides were washed and the excess was dried with absorbent paper; slides were mounted on laminula and glycerin in PBS (9:1).

In all steps, we prevented slide drying and tissue touching.

Slides were observed under dark field photomicroscopy with epi-illumination (Zeiss axioscan) with 490nm wavelength filter coupled to conventional photography camera. We executed photographic documentation of fields that presented different levels of marking (see below), enlarged 20 and 40 times, with exposure time of 30 or 60 seconds, depending on field light intensity. We used Pro-Image 100 film by Kodak. Upon developing it, photos were darkened so that images could look like the visualization under microscopy; however, it was not a pre-condition for appropriate assessment of photos.

### Assessment of the Technique

As to specificity: an extra slide of one of the cases was used as negative control, by omission of primary antibody.

The slide was blindly assessed by the author and the observer, together with the other slides of three more cases concerning the presence or not of labeling.

As to sensitivity: the reaction was applied to intestine of rat in total preparation, tissue with known presence of fibers and cell bodies containing VIP to test sensitivity of antibody. The sequence of amino acid of VIP was common for humans, rats and pigs, and the kit was applied to any of these species. The slides were assessed by an observer familiarized with this type of tissue and presence of labeling.

As to reproducibility of performance: the same technique was maintained from collection and manipulation of tissues for 8 cases (16 slides). We defined two parameters: with labeling and without labeling. We assessed the number of slides that presented labeling and out of the total of slides, we concluded the reproducibility of the technique.

**Figure 1:**
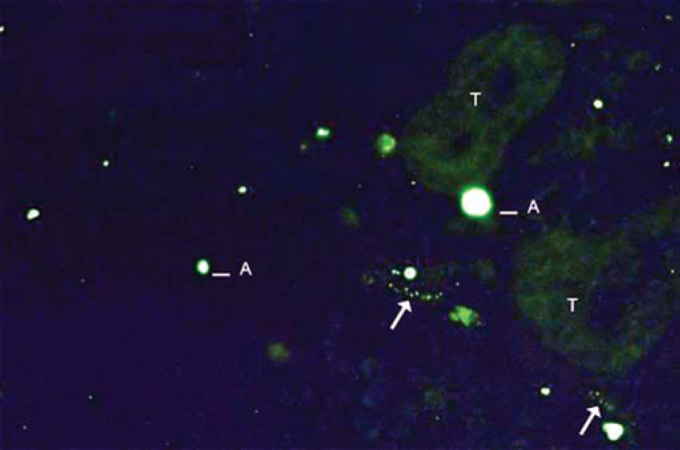
Example of labeling grade 1. Rare fibers identified (arrows). Artifact (A) and tissue auto-fluorescence (T).

**Figure 2:**
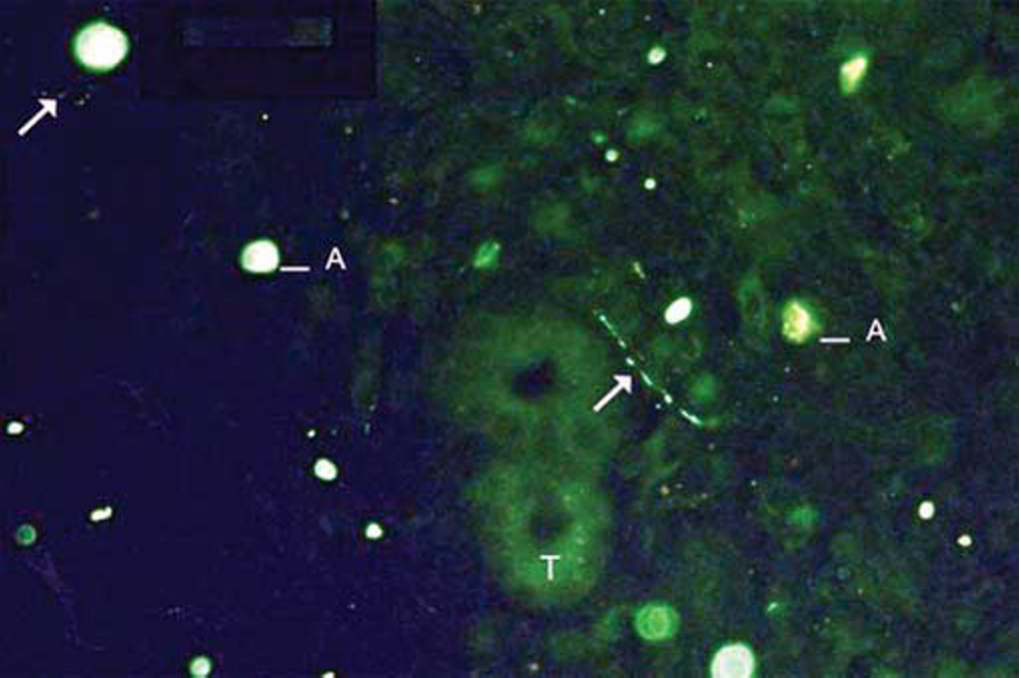
Example of labeling grade 2. Sparse fibers (arrows). Tissue auto-fluorescence (T) and artifacts (A).

**Figure 3:**
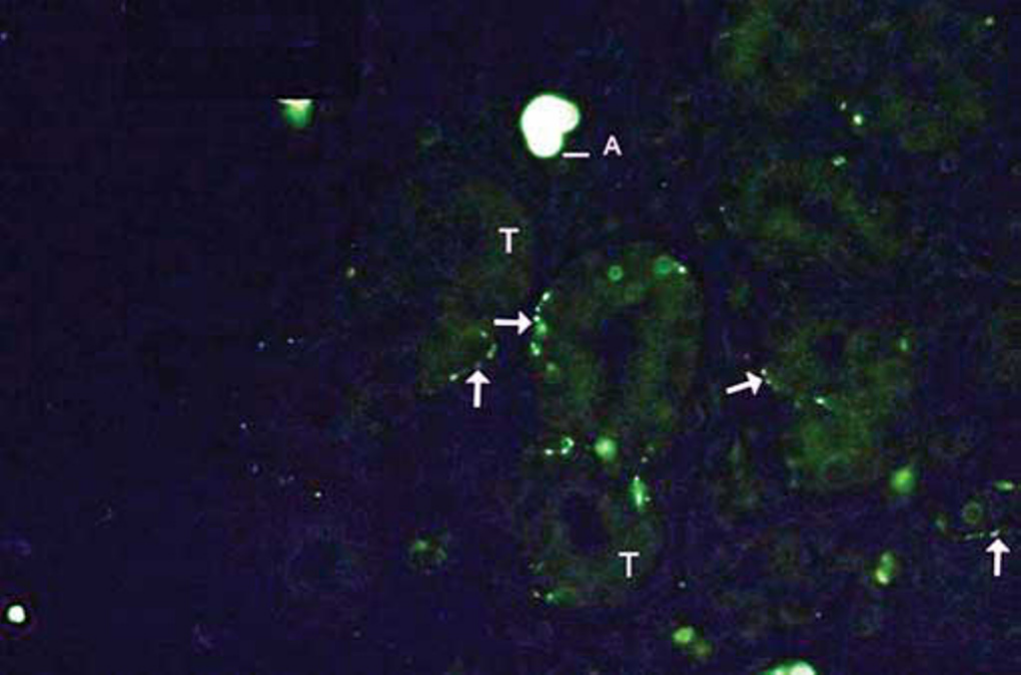
Example of labeling grade 3. Numerous fibers (arrows). Tissue auto-fluorescence (T) and artifacts (A).

**Figure 4:**
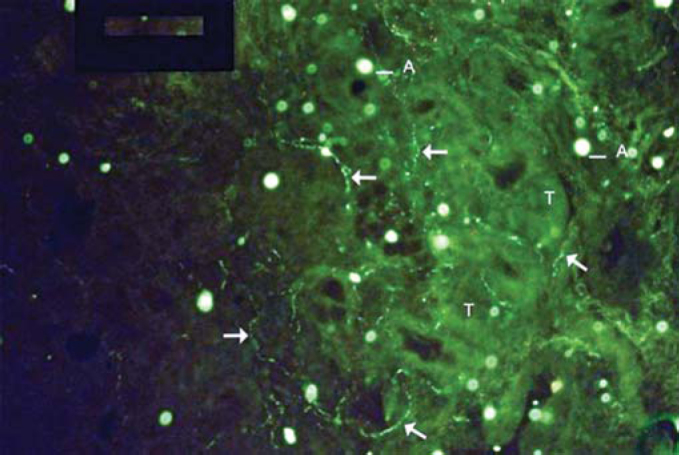
Examples of labeling grade 4. Many fibers (arrows), and it is possible to follow their pathway intertwined with glandular structures. More marked tissue auto-fluorescence (T) and artifacts (A).

As to reproducibility in interpretation of results: we defined standards of labeling grades concerning number of fibers labeled as follows: grade zero - no fiber in the field; grade one - rare fibers; grade two - sparse or few fibers; grade three - numerous fibers; grade four - many fibers. For each grade, we associated photos of fields to illustrate the classification (Figure 1, Figure 2, Figure 3, Figure 4).

The documentation produced photos of random microscopic fields that illustrated five grades of labeling. The total number of photos was 171 distributed by seven cases that presented labeling. The photos of the case in which there was no labeling were included for analysis. Five observers - A to E were presented to the examples that illustrated the grades of labeling, oriented towards their meaning and required to classify photos independently. Three observes were Ph.D. professors, Department of Morphophysiology Sciences, UEM, experienced in immunofluorescence in animal tissues and two were taking post-graduation studies in Pharmaceutical Sciences, without experience in the topic. The author was the sixth observer F.

The grades of labeling attributed by the six observers were compared concerning coincidence or discrepancy of results using Kruskal-Wallis test for non-dependent variables.

## RESULTS

As to specificity: negative control did not show labeling after blind assessment of 2 observers.

As to sensitivity: the method showed labeling when applied to total preparation of rat intestine VIP, proving to be sensitive to VIP.

As to reproducibility of execution: out of 16 slides, 14 showed VIP labeling (87.5%).

As to reproducibility of interpretation of results: grades attributed to photos by the 6 observers were compared using Kruskal-Wallis test, and it showed that there were differences among the interpretation of observers (p < 0.05). Observer E differed from A, B and D, but not from C and F (Table 1). Considering the other five observers, there was uniformity of interpretation of results.

**TABLE 1:** Result of Kruskal-Wallis test demonstrating difference between interpretation of observer E and interpretations of observers A, B and D (p < 0.05).

	A	B	C	D	E	F
A		1,0000	1,0000	1,0000	0,0043	0,1507
B	1,0000		1,0000	1,0000	0,0459	0,8431
C	1,0000	1,0000		1,0000	0,5085	1,0000
D	1,0000	1,0000	1,0000		0,0204	0,4746
E	0.00433	0.0459	0,5085	0,0204		1,0000
F	0,1507	0, 8 431	1, 0 000	0, 4 746	1, 0 000	

**Graph 1:**
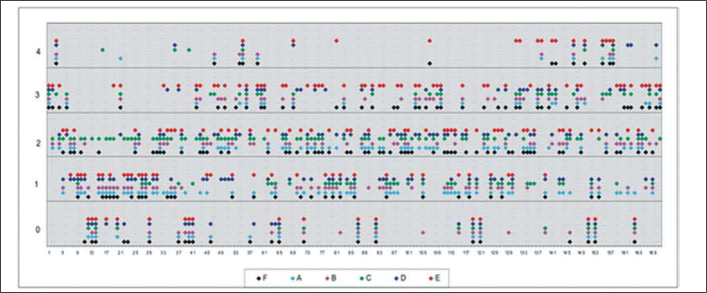
Distribution of grades (axis Y) attributed to photos (axis X) for each observer (color points).

Distribution of grades attributed to each photo by the observers can be assessed in Graph 1.

## DISCUSSION

The review presented here shows an unquestionable importance to neuropeptides in human nasal mucosa concerning physiology and pathophysiology of diseases. Nasal innervation deeply participates in physiological phenomena related to reflexes of defense of airways. In addition to classical neurotransmitters, much has been studied about neuropeptides. Some of the general characteristics differentiate them from classical neurotransmitters, among them the fact that their removal occurs by synthesis at the level of cell body, and not at reuptake of synaptic chink, meaning that their effects are less dramatic^5^. Thus, they are targets for research studies with therapeutic purposes, since neurotransmitters antagonists with these characteristics tend to have fewer side effects^4^.

Both sensorial innervation and sympathetic and parasympathetic innervation contain neuropeptides in nasal mucosa whose actions have been investigated.

Once we confirm the importance of neuropeptides in nasal physiology and pathophysiology, it is interesting that this knowledge develops and that concepts are solidified. Therefore, the higher the number of research studies on the topic, the faster the knowledge about neuropeptides in human nasal mucosa could point to new clinical perspectives.

Actions of neuropeptides in respiratory system, specifically the nasal mucosa, have been studied through in vivo and in vitro experiments, both in animals and in humans, as exemplified in the studies that follow.

The presence of neuropeptides in nervous fibers with morphological fidelity is only performed by using immunelabeling techniques. Quantitative methods do not provide morphological information. In turn, immunelabeling is difficult to quantify, meaning it provides mainly qualitative information. The investigation of neuropeptides in nasal mucosa, both quantitative and qualitative, brings information about nasal physiology and pathophysiology. Comparing normal subjects and allergic rhinitis patients using these techniques has added much to the understanding of the topic^3^. Upon proceeding in this field, we were faced by a considerable barrier from the technical perspective, which is characterized by the reaction of immunelabeling. The literature on rhinology shows the methodology in a rather simplistic way, omitting aspects that we later realized to be important, in addition to many variations in technical details among the studied papers. From collection of nasal mucosa we can detect paucity of information in the applied literature. Some rare authors described the technique, normally simply alluded as collection^1^, ^2^, ^3^, ^15^, ^22^.

Aspects concerning topical vasoconstriction, infiltration, luxation of concha and material are not described as a routine. Since nasal mucosa is an extremely reactive tissue to stimuli, leading to release of neuropeptides from its fibers, special attention was given to the collection procedure during nasal surgery, because it could have influenced the results. From chemical stimulus, which in theory would lead to degranulation and elimination of neuropeptides, we took care to avoid use of anesthetic gases in mask during induction of general anesthesia^5^. After attempts to collect fragments of nasal mucosa without vasoconstriction, in an attempt to minimize the stimuli, we observed that the procedure was hindered by deficit in visualizing the operation field, because of bleeding, which could result in excessive mechanical manipulation and fragmentation of tissue, not desirable in the studied technique. We came to the conclusion that we should apply vasoconstriction, in addition to medial luxation of concha. The collection was made at the beginning of the surgery and not after it, because excessive manipulation would have already been made, as well as the need for infiltration.

The fixation methods used, as well as time, also varied in the literature, and mixed fixation solutions were the most frequently employed^8^, ^24^. Special attention was directed to time of fixation, which was longer, despite the better preservation of the tissue, and the risk of loss of antigenicity, which in turn, also increased^25^. Fixation procedures were followed according to a study published, with some adaptations^6^: longer time (six hours) to allow complete penetration of fixation solution and maintenance of 4°C to limit excessive fixation with risk of loss of antigenicity. Once we obtained good tissue fixation, confirmed by observation of slides stained with HE and presence of immunofluorescence labeling, the fixation method was considered to be appropriate.

The fact that the tissue presented auto-fluorescence with the employed fixation solution^21^, it allowed identification of the nasal mucosa components, such as glands and vessels, creating an advantage.

The ideal storage method for immunelabeling is freezing^23^. We studied different techniques, despite not detailed, and storage temperatures described in the literature^3^, ^22^. Storage temperature of -70°C was chosen in an attempt to better preserve the tissue, considering fragility of neuropeptides.

We also observed freezing with soaked specimen for section with cryostat, which facilitated the procedure, sometimes hindered by dryness of tissues.

Different thickness is alluded in reference studies on immunofluorescence. Wider sections, larger than 10µm, allow follow up of fibers for a longer path, facilitating identification and making the results more understandable and rich.

Photomicroscopic documentation of nervous fiber immunofluorescence requires great sensitivity and resolution. Conventional photography perfectly met these requirements, given that digital equipment with these characteristics is extremely expensive.

We noticed differences between the limited vision of ENT physicians and researchers' visions, leading to unclear descriptions, which seemed to have taken an apparently satisfactory methodology. To confirm this impression, we proposed a method assessment concerning specificity, sensitivity, reproducibility of performance and of results' interpretation.

Results showed that the method is effective because the negative control did not show labeling. Immunelabeling is a specific method because it is based on antigen-antibody reaction. However, knowledge and familiarity with tissue morphology are required to interpret the immunofluorescence images of nervous fibers. We observed background fluorescence and artifacts by fluorescein debris; any observer not very involved with the topic may produce too many false-positive results.

Sensitivity of the laboratory equipment was tested in positive control of rat intestine, a tissue rich in VIP, including cell bodies. Once we confirmed efficiency of method in the tissue, it is reasonable to state that it is sufficiently sensitive to interpret the results in human nasal mucosa. However, we can not state that all VIP present in in vivo tissue was demonstrated by the method, because the different steps from collection, fixation and storage included factors that could have led to reduction of its presence (degranulation and enzymatic degranulation) or to loss of antigenicity. There is no gold standard to have it compared with.

Reproducibility of execution was considered satisfactory and the technical detailing presented may contribute to new experiments.

The significant difference in grades attributed by photos observed by examiners reveals the need to have morphological knowledge of how fibers are presented by immunofluorescence. Only one observer - a post-graduation researcher- had opinions different from the others. Despite this fact, none of the observers had any experience in specific interpretation of human nasal mucosa. There were three researchers that worked with immunelabeling of neuropeptides in animal tissues, which in general contain nerves, ganglions and cell bodies, presenting a different pattern of labeling. The two others were taking post-graduation studies, including the one whose interpretation differed from the others. The interpretation of observer F, or the author, was more completed considering that in addition to the photos, he had observed the slides under microscope, which inevitably presents the images with more details, in addition to constituting a dynamic analysis. Moreover, the author was the most involved observer, who had theoretical conditions to interpret the images. Individually, none of the 5 observers differed from the author.

Considering these aspects, we deducted that the method is reproducible in its interpretation of results provided that there is familiarity with fiber labeling aspects present in the nasal mucosa.

## CONCLUSION

The immunofluorescence method proposed for VIP in human nasal mucosa is sufficiently specific, sensitive and reproducible, considered to be useful in investigations on neuropeptides in human nasal mucosa.
